# Enhanced wound regeneration by PGS/PLA fiber dressing containing platelet-rich plasma: an in vitro study

**DOI:** 10.1038/s41598-024-62855-w

**Published:** 2024-05-26

**Authors:** Parisa Heydari, Anousheh Zargar Kharazi, Laleh Shariati

**Affiliations:** 1 Department of Materials Engineering, Isfahan University of Technology, Isfahan, 84156-83111 Iran; 2 Applied Physiology Research Center, Isfahan, Cardiovascular Research Institute, Isfahan University of Medical Sciences, Isfahan, Iran; 3 Biomaterials Nanotechnology and Tissue Engineering Faculty, School of Advanced Technologies in Medicine, Isfahan University of Medical Sciences, Isfahan, Iran; 4 Biosensor Research Center, Isfahan University of Medical Sciences, Isfahan, Iran

**Keywords:** Skin tissue engineering, PRP, Angiogenesis, Electrospinning, Macrophage polarity, Biomaterials, Biomedical engineering

## Abstract

Novel wound dressings with therapeutic effects are being continually designed to improve the wound healing process. In this study, the structural, chemical, physical, and biological properties of an electrospun poly glycerol sebacate/poly lactide acid/platelet-rich plasma (PGS/PLA-PRP) nanofibers were evaluated to determine its impacts on in vitro wound healing. Results revealed desirable cell viability in the Fibroblast (L929) and macrophage (RAW-264.7) cell lines as well as human umbilical vein endothelial cells (HUVEC). Cell migration was evident in the scratch assay (L929 cell line) so that it promoted scratch contraction to accelerate in vitro wound healing. Moreover, addition of PRP to the fiber structure led to enhanced collagen deposition (~ 2 times) in comparison with PGS/PLA scaffolds. While by addition PRP to PGS/PLA fibers not only decreased the expression levels of pro-inflammatory cytokines (IL-6 and TNF-α) in RAW-264.7 cells but also led to significantly increased levels of cytokine (IL-10) and the growth factor (TGF-β), which are related to the anti-inflammatory phase (M2 phenotype). Finally, PGS/PLA-PRP was found to induce a significant level of angiogenesis by forming branching points, loops, and tubes. Based on the results obtained, the PGS/PLA-PRP dressing developed might be a promising evolution in skin tissue engineering ensuring improved wound healing and tissue regeneration.

## Introduction

Skin tissue is an important organ for protecting and maintaining body homeostasis^1^. It is essential for skin wounds caused by burns, physical injuries, and diabetics or other diseases to be treated quickly. “Hard-to-heal” damages or nonfunctional scars can result from chronic wounds caused by insufficient angiogenesis, decreased collagen deposition, extended immune response, and other abnormalities. Therefore, in order to speed up wound healing for chronic skin injuries, bioactive dressings must be used^2^. The wound healing is a dynamic process driven by different biological signals and mediators and in constant need of optimization^3^. However, the process might be dysregulated by such events as prolonged inflammation, insufficient angiogenesis, and infection, giving rise to the so called “hard-to-heal wound”^4^. The situation becomes even worse with large skin defects that pose an essential challenge to clinical wound treatment. During wound healing, angiogenesis at the wound site plays a critical role in effective wound repair^5^. It is well established that proliferating capillaries not only bring oxygen and nutrients to the new tissue being regenerated but also remove metabolized waste products from the granulation tissue. Inadequate angiogenesis will, therefore, result in the necrosis of the wounded tissue, induced hypoxia, and proliferation of anaerobic bacteria. Recently, various wound dressings has been recently investigated and developed for accelerating and improving wound healing^6^.

Biomaterial wound dressings available in the various forms of hydrogels, membranes, and scaffolds have been widely used in recent years for their advantageous properties such as cell viability, biodegradation, controlled drug or biological agents delivery, accelerated angiogenesis, and faster wound healing process^7^. For example, Poly Glycerol Sebacate (PGS) is one of attractive polymers for wound dressing application. PGS is an ester-bond elastomer formed by poly condensation of poly alcohols containing multiple hydroxyl groups and dicarboxylic acid^8^. Being an elastomer, PGS enjoys not only flexible mechanical properties but also offers cell viability and biorestorability (i.e., surface degradation)^9^. These unique properties have encouraged both in vitro and in vivo studies of PGS focused on applications in heart tissues^10^, blood vessels^11^, cartilage^12^, nerve guide^13^, and skin tissues as well as their use as potential drug carriers^14^. On the other site, Poly Lactide Acid (PLA) is another synthetic, biodegradable, and biocompatible polymer that dissolves in most solvents and interacts with most hydrophilic materials^15^. This polymer with ideal mechanical properties have various applications in skin tissue engineering, cardiovascular tissue engineering, and controlled drug delivery^16^.

Wound dressings have been manufactured using a vast variety of techniques, with electrospinning regarded as the ideal manufacturing candidate compared to freeze-drying, solvent casting, particle leaching, injection molding, and phase separation^17^. Electrospun fibers offer an increased surface area to volume ratio that makes it attractive for developing biomimetic scaffolds with a high porosity whereby the ECM architecture thus provided promotes cell adhesion^18^. Electrospinning of PGS can be difficult because of such drawbacks as low viscosity, low glass transition temperature (Tg), and low conductivity^19^. Hence, PGS cannot be electrospun in the absence of other conductive materials or polymers. To address this problem, Poly(Caprolactone) (PCL)^14^, gelatin^20^, Poly (Vinyl alcohol) (PVA)^21^, and Poly (lactide)^22^ have been used as the carrier polymers in manufacturing PGS fibers.

Despite significant advances in PGS and PLA for wound healing application, some issues still inevitably remain. For example, it is necessary for wound dressing to have ideal biological properties to accelerate wound healing process. Growth factors (GFs) have demonstrated important roles in angiogenesis, tissue regeneration, and anti-inflammatory wound healing processes. Notably, clinical practice has confirmed that the synergism of many growth factors, as opposed to the individual combat of a single GF, is a potent treatment strategy. However, the formulation of various GFs is still challenging, and the high costs are unaffordable^23^. Platelet-rich plasma (PRP) is a plasma concentrate that has been isolated from whole blood. It has been identified as a regenerative agent that facilitates wound healing because of the “cocktail” effect produced by a range of GFs such as vascular endothelial growth factors (VEGF), platelet-derived growth factors (PDGF), epidermal growth factors (EGF), and transformed growth factor-β (TGF-β). Therefore, in order to ensure that PRP has the desired therapeutic benefits, advanced dressing platforms are essential. In previous studies, a combination of PGS and PLA polymers has been investigated to fabricated an ideal wound dressing. On the other hand, recently, PRP was widely used in the wound healing process. For this reason, a combination of PGS/PLA and PRP is being studied for the first time.

As shown in Schematic 1, in this study considering the physical properties of PGS/PLA and the biological properties of PRP, the novel wound dressing based on PGS/PLA-PRP was preparation by using electrospinning technology and examined the physical, chemical, and various biological activity such as cell migration, angiogenesis, expression Collagen I gene and inflammatory responses for wound healing applications.

**Schematic 1:**
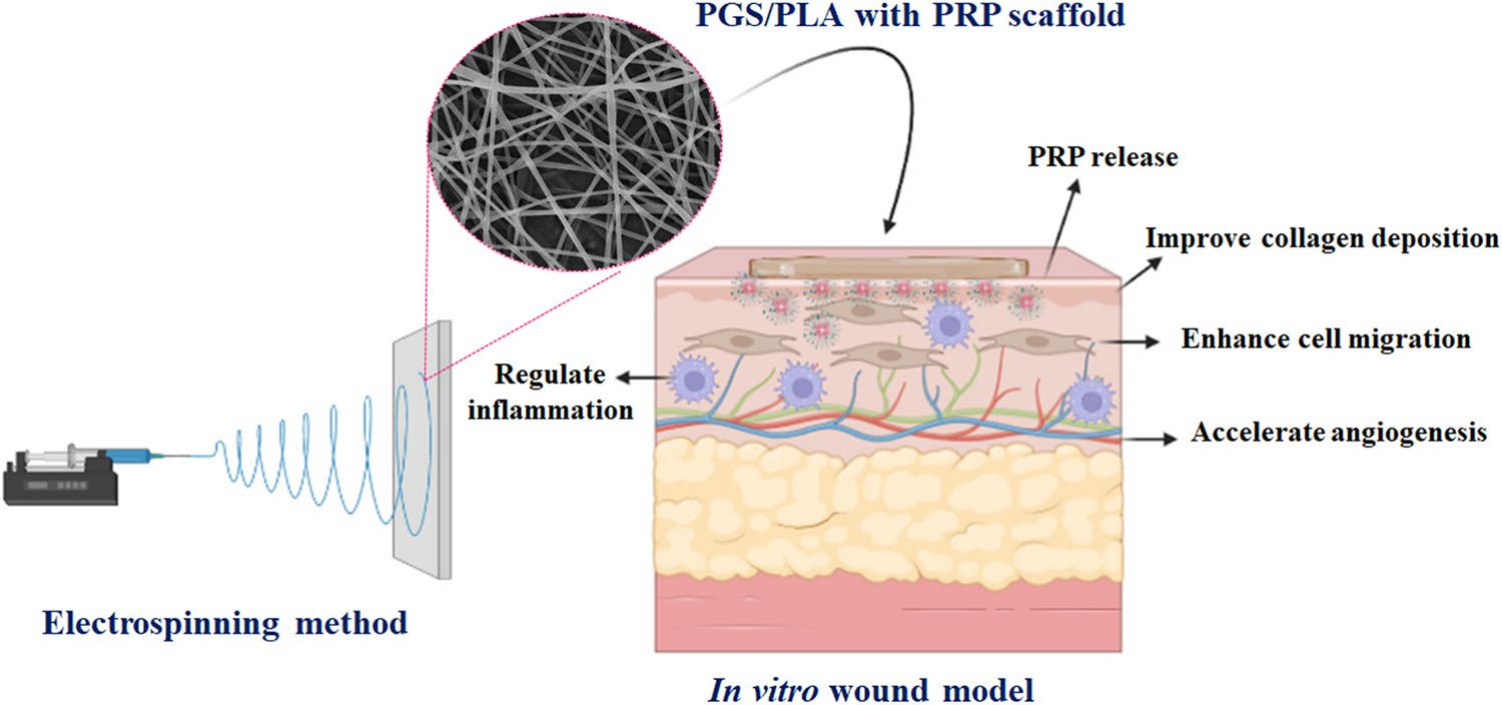
The schematic illustration of the design of a suitable wound dressing such as PGS/PLA-PRP with desired angiogenesis, anti-inflammatory response, collagen deposition, and cell proliferation.

## Materials and methods

### Materials

In this study, Sebacic acid (99%, Merck, Germany), Glycerol (99%, Merck, Germany), Poly Lactic Acid (PLA) (Mw = 200 kDa, Sigma-Aldrich), chloroform (Sigma-Aldrich), Dimethylformamide (DMF) (Merck, Germany), dimethyl thiazole diphenyltetrazolium bromide (MTT, Sigma-Aldrich), sodium citrate (Vacuette, Switzerland), Lipopolysaccharide (LPS, Sigma-Aldrich), dimethyl sulfoxide (DMSO) (Merck, Germany), Dulbecco’s Modified Eagle Medium (DMEM-high and F12-DMEM), fetal bovine serum (FBS), streptomycin, penicillin (Bioidea, Iran), and phosphate buffered saline tablets (PBS, Sigma-Aldrich) were used.

### PGS synthesis

PGS was synthesized as described in the literature^24,25^. Briefly, the PGS polymer was synthesized via condensation polymerization using a 1:1 mixture of sebacic acid and glycerol heated at 120 °C for 24 h under the N_2_ atmosphere and kept at 40 °C under vacuum for another 24 h to obtain a white and viscous PGS polymer.

### Preparation of platelet-rich plasma (PRP)

The present study design was approved by the Ethical Committee of Isfahan University of Medical Sciences (Ethic Number: IR.MUI.RESEARCH.REC.1399.695). Peripheral blood samples were collected from healthy volunteers that informed consent was obtained from all participants. Peripheral blood concentrated in 9 ml tubes containing 3.8% sodium citrate. Following the first spin at 200×*g* for 10 min, the liquid supernatant was transferred to another tube in order to run the second spin at 2500×*g* for 15 min. The upper two-thirds of the cell-free supernatant was subsequently removed and the platelet pellet (platelets count ~ 5–6 × 10^6^/ul) was suspended in the lower one-third of the plasma to obtain PRP^26^. In this study, to avoid the loss of efficacy, we used fresh PRP under 3 h after preparation. Although, for clinical applications PRP can be stored at − 80 °C for 1 month or in liquid nitrogen for 6 months to preserve its characteristics^27^.

### Preparation of electrospun PGS/PLA-PRP fibers

As a first step, PLA solution was prepared at a concentration of 5% (w/v) in a solution of chloroform and DMF solvent with an aspect ratio of 8:2 for 1 h. Afterwards, PGS was dissolved for 30 min in the same solvents at a concentration of 10% (w/v) followed by 15 min of mixing the PLA and PGS solutions thus obtained. To prepare the PGS/PLA-PRP solution, 0.5% (w/v) (0.75 mg in 1 ml) of PRP was added to the PGS/PLA solution and mixed for 10 min before the PGS/PLA and PGS/PLA-PRP membranes were fabricated by electrospinning. To prepare scaffolds, the fibers were electrospun at a feed rate of 0.5 ml*/h* using a needle 0.6 mm in diameter, a collector-to-needle distance of 18 cm, and an applied voltage of 20–25 kV. In this study, it can be said that the electrospinning process of a 1 ml solution took almost 2 h and the dimensions of the electrospun scaffold are usually 5 cm^2^.

### Materials characterization

#### Chemical structure and morphological evaluation

Fourier transform infrared spectroscopy (FTIR) was used to evaluate the chemical bonds and functional groups of the scaffolds. IR beam absorption peaks were observed at wavelengths between 400 and 4000 cm^−1^.

#### Microstructure and morphology

Scanning electron microscopy (SEM, Philips, XL30) was used to evaluate fiber morphology, fiber diameters, and porosity^28^. In this process, the samples were sputtered with gold before SEM imaging^29^. Using the Image-J Software (Version 1.52v, USA), 25 fibers were randomly selected to determine average fiber diameter. Also, porosity was evaluated in MATLAB^30^.

#### Evaluation of wettability, swelling and degradation

Using water contact angle evolution, the water wettability of the PLA, PGS/PLA, and PGS/PLA-PRP scaffolds of 1 cm^2^ dimension at room temperature (n = 3) was assessed.

The swelling ratio of electrospun specimens (1 cm^2^) was evaluated by immersion in 5 ml of the PBS solution (37 °C and pH 7.4) for 24 h after they had been weighed^31^. Swelling ratio was subsequently calculated using Eq. (1):

1 $$ {\text{Swelling ratio }}\left( \% \right) \, = \, \left( {{\text{W}}_{2} - {\text{W}}_{1} } \right)/{\text{W}}_{1} \times 100, $$

where W_2_ and W_1_ represent specimen weights in the swollen and dry states, respectively.

To evaluate in vitro degradation rate, scaffolds (1 cm^2^) were initially weighed and then immersed in a 10 ml PBS solution (37 °C and pH 7.4) for 1, 3, 5, 7, 14, 21, and 28 days^31^. After removal from the PBS solution, the specimens were dried and weighed again. Degradation rate was calculated using Eq. (2) below:

2 $$ {\text{Degradation ratio }}\left( \% \right) \, = \, \left( {{\text{W}}_{1} - {\text{W}}_{2} } \right)/{\text{W}}_{1} \times 100, $$

where W_2_ represents specimen’s weight after degradation in time points and W_1_ represents its weight before soaking in PBS.

#### PRP release behavior

PRP release is a technique for measuring protein concentrations (such as PRP proteins) in solutions. For this purpose, membrane specimens (1 cm^2^) were used. Briefly, Bovine Serum Albumin (BSA) was used as the standard sample to prepare 25 µg/ml of the BSA stock solution. The standard curve was obtained by adding reagent A (Coomassie brilliant blue G-250 dye) to each concentration of the stock solution (20 µl reagent A + 80 µl standard solution). Then, 10 µl of each time point of the sample solution was transferred to a well and diluted with 80 µl of water. As a final step, 20 µl of reagent A was added to the wells, incubated for 5 min, and the proteins were reacted. Using the standard curve, the concentrations of the proteins released were determined during 24 h. Finally, absorbance was read at 595 nm using an ELISA reader (Biorad-USA)^32,33^.

### In vitro cell assay

#### Cell viability

MTT assay was performed to evaluate the cell viability of the wound dressing membranes (1 cm^2^) with and without PRP. For this purpose, membrane specimens were sterilized for 20 min in 60 v/v% ethanol followed by 1 h of exposure to UV light. Murine fibroblast cell line (L929), macrophage cell line (RAW264.7), and human umbilical vein endothelial cells (HUVEC), all procured from Cell Bank at Pasteur Institute—Iran, were used for cell viability determinations. All the three cell lines were incubated in DMEM containing 10 v/v% FBS and 1 v/v% streptomycin/penicillin at 37 °C in 5% CO_2_. Subsequently, 10^4^ cells/well were seeded on both the specimens and the tissue culture plate (TCP) used as control. The specimens with cells were then incubated for 5 days while the culture medium would be changed every 2 days.

To investigate cell viability via MTT assay, the culture medium was discarded at days 1, 3, and 5. Subsequently, the cell-seeded specimens and the control were incubated in 100 μl of the MTT solution (5 mg/ml) for 3 h. Once the dark blue formazan crystals had been dissolved in DMSO, 100 μl of the dissolved formazan solution of each specimen was transferred to the 96-well plate and optical density (OD) was read at 490 nm using an ELISA reader (Biorad-USA). Finally, relative cell survival was calculated using Eq. (3) below:

3 $$ {\text{Relative cell survival }}\left( {\% {\text{ control}}} \right) \, = \frac{{{\text{X}}_{{\text{S}}} - {\text{X}}_{{\text{d}}} }}{{{\text{X}}_{{\text{t}}} - {\text{X}}_{{\text{d}}} }} \times 100, $$

where X_S_, X_d_, and X_t_ denote absorbance of the specimen, DMSO as the blank specimen, and TCP as the control, respectively.

### Scratch test (in vitro wound healing)

In order to evaluate cell migration and in vitro wound healing (scratch assay), L929 cells (3 × 10^4^ cells/well) were seeded in 24 well-plates as a model system and incubated for 24 h at 37 °C in 5% CO_2_ to permit cell adhesion and monolayer formation. Following previous studies^34^, in vitro wound models were made using a sterile 100 µl pipette tip on the middle of a confluent surface using specimens 10 mm in diameter placed on both sides of the scratch. The closure of in vitro wounds was observed using an optical microscope at 0, 16, and 24 h while the cell-containing TCPs were used as the control. Wound healing was evaluated by ImageJ Software (2019, MRI wound healing tool) and reported in percents at each specified time based on the following equation:

4 $$ {\text{Wound healing }}\left( \% \right){ } = \frac{{{\text{A}}_{0} - {\text{ A}}_{{\text{t}}} }}{{{\text{A}}_{0} }} \times 100, $$

where *A*_*t*_ and *A*_0_ are wound area at time *t* and initial area of the specimen, respectively.

#### Collagen I gene expression

L929 fibroblast cells (10^4^ cells/ml) were seeded on the films in a 12-well plate and cultured for 24 h to evaluate Collagen I expression at RNA level. At the time point, total RNA was extracted using the RNA extraction kit. This was followed by synthesizing cDNA from 1 mg RNA using a cDNA Synthesis Kit. Finally, Quantitative Real Time PCR were performed using StepOnePlus real-time polymerase chain reaction (PCR) (AppliedBiosystems, USA). *Beta 2 Microglobulin* (B2M) was used as a housekeeping gene to normalize RNA expression levels. Finally, the Livak method was used to evaluate the Fold change^35^. The sequences of the primers are listed in Table 1.

**Table 1:** Specific primers for real-time PCR.

Collagen I-F	CCAGAACATCACCTATCACTGG
Collagen I-R	AAGTTCCGGTGTGACTCGTG
B2M-F	GGTCTTTCTGGTGCTTGTCTC
B2M-R	TCCCGTTCTTCAGCATTTG

#### Inflammatory response

In order to evaluate inflammation factor, scaffolds with and without PRP (1 cm^2^) were seeded with RAW-264.7 macrophages at a seeding density of 10^6^ cells/well followed by stimulation with LPS (100 ng/ml) for 24 h. The cell-containing supernatant was subsequently harvested and the levels of tumor necrosis factor-α (TNF-α), interleukin-6 (IL-6), interleukin-10 (IL-10), and tissue growth factor-β (TGF-β) were measured using enzyme-linked immunosorbent assay kits (KPG, Iran)^36^.

#### In vitro angiogenesis

Matrigel-based models were used to evaluate angiogenic activity as determined by angiogenesis stimulators. Tube formation was measured in 24-well plates coated with Matrigel (Sigma, Germany) at 37 °C. After trypsinization, HUVEC cells at passage 5 were seeded onto Matrigel^37^. Similarly, conditioned medium-electrospun specimens (the extract of 1 cm^2^ of electrospun samples in DMEM medium during 48 h in 37 °C) were added to the wells at the same time point. Tube formation was evaluated after 24 h using an inverted microscope. At each specified time point, the total branching points, total tube length, and total loops were quantified using the Image J software (2019, Fiji vessel analysis tool).

### Statistical analysis

In this study, all the measurements were repeated three times (n = 3) and the data thus obtained were subjected to the one-way ANOVA test and the Tukey–Kramer post-hoc test using Graph Pad Prism Software (V.9) to evaluate the data at a statistical significance level of < 0.05.

### Ethics statement

All procedures were conducted according to relevant guidelines and regulations, and all experiments were conducted in accordance with standard guidelines.

## Results and discussion

### Fabrication and characterization of PGS/PLA-PRP scaffolds

**Figure 1:**
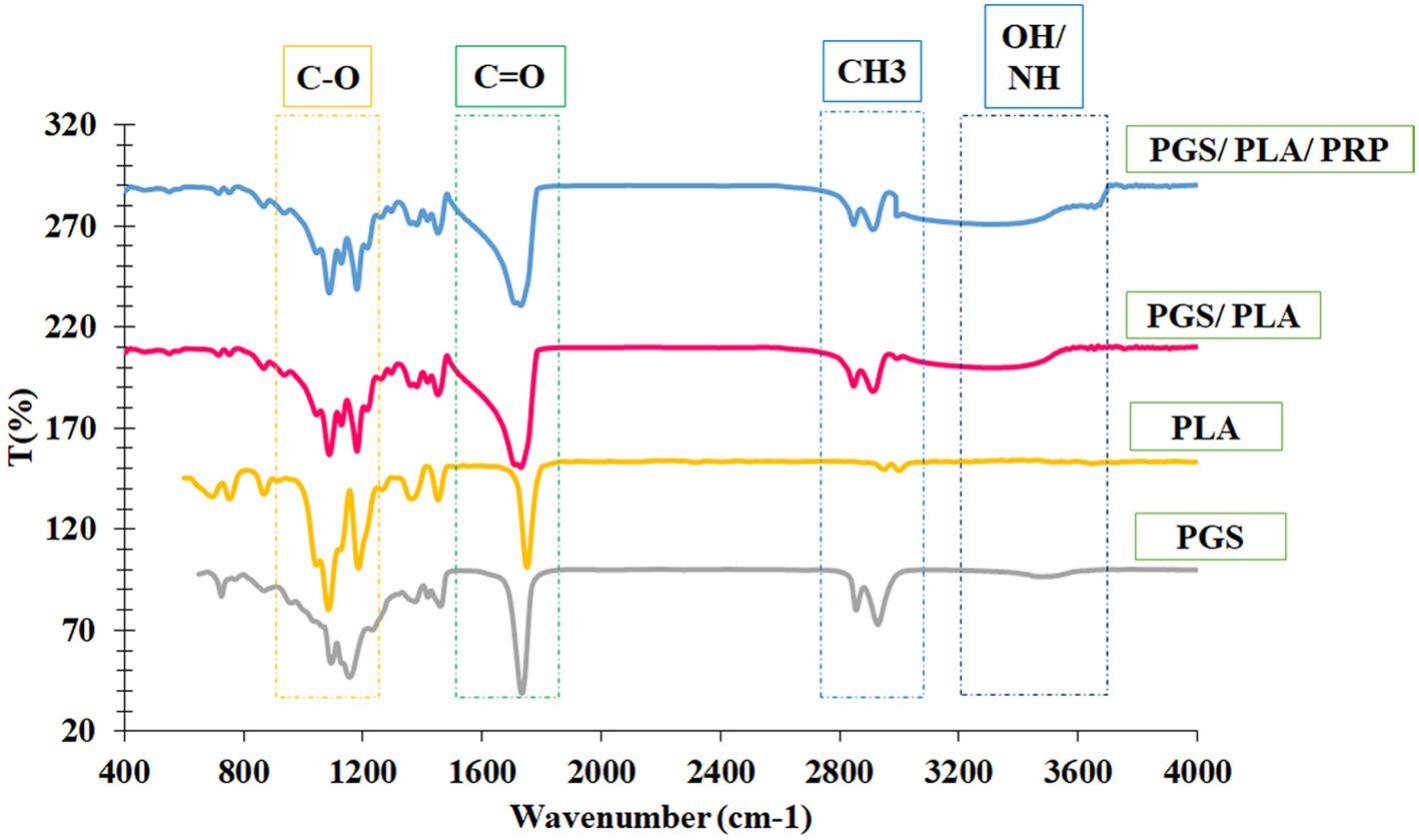
FTIR spectra of PGS, PLA, electrospun PGS/PLA, and PGS/PLA-PRP scaffolds.

The FTIR spectra of PGS, PLA, PGS/PLA, and PGS/PLA-PRP are presented in Fig. 1. The peaks around 2925 cm^−1^ and 2853 cm^−1^ were related to the methyl and alkane groups of the PGS polymers while that around 3458 cm^−1^ belonged to the hydroxyl group of two polymers and the ones at around 1171 cm^−1^ and 1731 cm^−1^ belonged to the C–O and C=O functional groups related to PGS^20,30^. Furthermore, the peaks around 753 cm^−1^ (C=O bending bond), 1088 cm^−1^ (C–O stretching bond), and 2292 cm^−1^ (hydroxyl group) belonged to PLA^22^. The peak around 3046 cm^−1^ (C–H stretching group) in the PLA polymer overlapped with the broad and wide peak of PGS, OH groups (3458 cm^−1^). Obviously, these characteristic peaks demonstrated that the PGS and PLA polymers had been blended in the PGS/PLA electrospun scaffold. An FTIR spectrum of PGS/PLA-PRP confirmed the presence of N–H from PRP (protein) in the 3500 cm^−1^ area^38^.

### Microstructure and morphology

**Figure 2:**
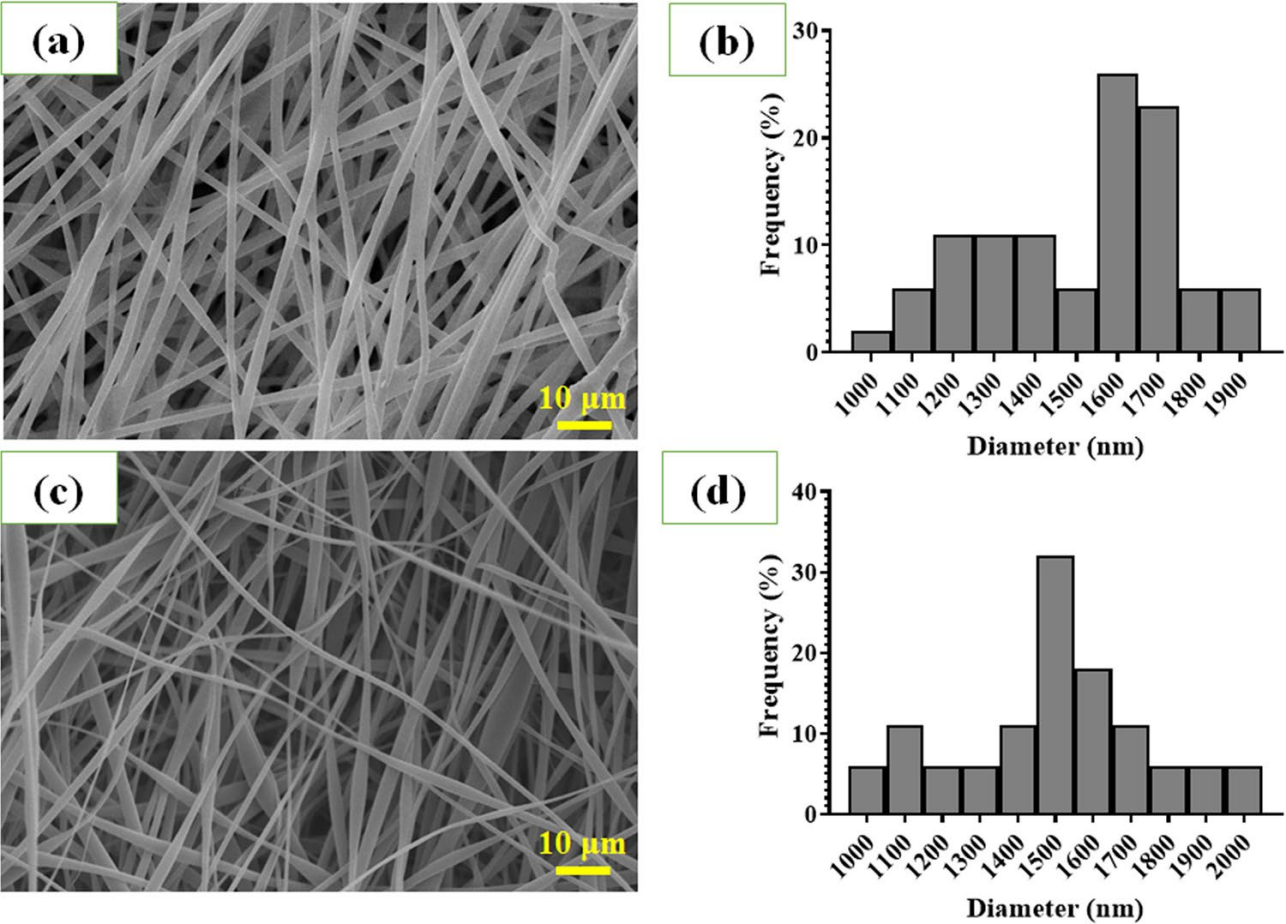
Evaluation of the morphology and distribution of fiber diameters: (**a**) SEM image of PGS/PLA, (**b**) fiber distribution histogram of PGS/PLA, (**c**) SEM image of PGS/PLA-PRP, and (**d**) fiber distribution histogram of PGS/PLA-PRP.

The surface morphologies of PGS/PLA and PGS/PLA-PRP fibers are shown in Fig. 2. As can be seen, the PGS/PLA (Fig. 2a) and PGS/PLA-PRP (Fig. 2c) fibers exhibit a regular, uniform, and porous network morphology. The diagram of the fiber diameter distribution was also provided by measuring the diameters of 20 fibers in the SEM images (Fig. 2b,d) to find that the average fiber diameters for PGS/PLA and PGS/PLA-PRP scaffolds were 1632 ± 298 nm and 1565 ± 411 nm, respectively (p > 0.05). SEM results demonstrated that PRP loading on the scaffold had no negative effects on fiber shape, morphology, or size. Moreover, average porosities of 83.2 ± 4.2% and 94.55 ± 3.8% were measured for PGS/PLA and PGS/PLA-PRP wound dressing membranes, respectively. In fact, adding PRP to the fiber structure increased pore connectivity and, thereby, porosity. It could be because adding PRP to a polymer solution alters its viscosity, surface tension, and interfacial tension between its PGS and PLA components. Pore size and porosity may be affected by each of these variables^39^. The PRP-containing samples lead to improved cell adherence on the surface due to their much greater pore size and porosity value compared to PGS/PLA scaffold. On the other word, Membrane porosity is a decisive parameter in the wound healing process due to its impacts on permeability and distribution of nutrients and oxygen across the wound area^40^. Previous study indicates a surface porosity of around 85% as a desirable level for skin tissue engineering applications since it provides an ideal surface for skin cell interactions^16^.

### Evaluation of wettability, swelling and degradation

**Figure 3:**
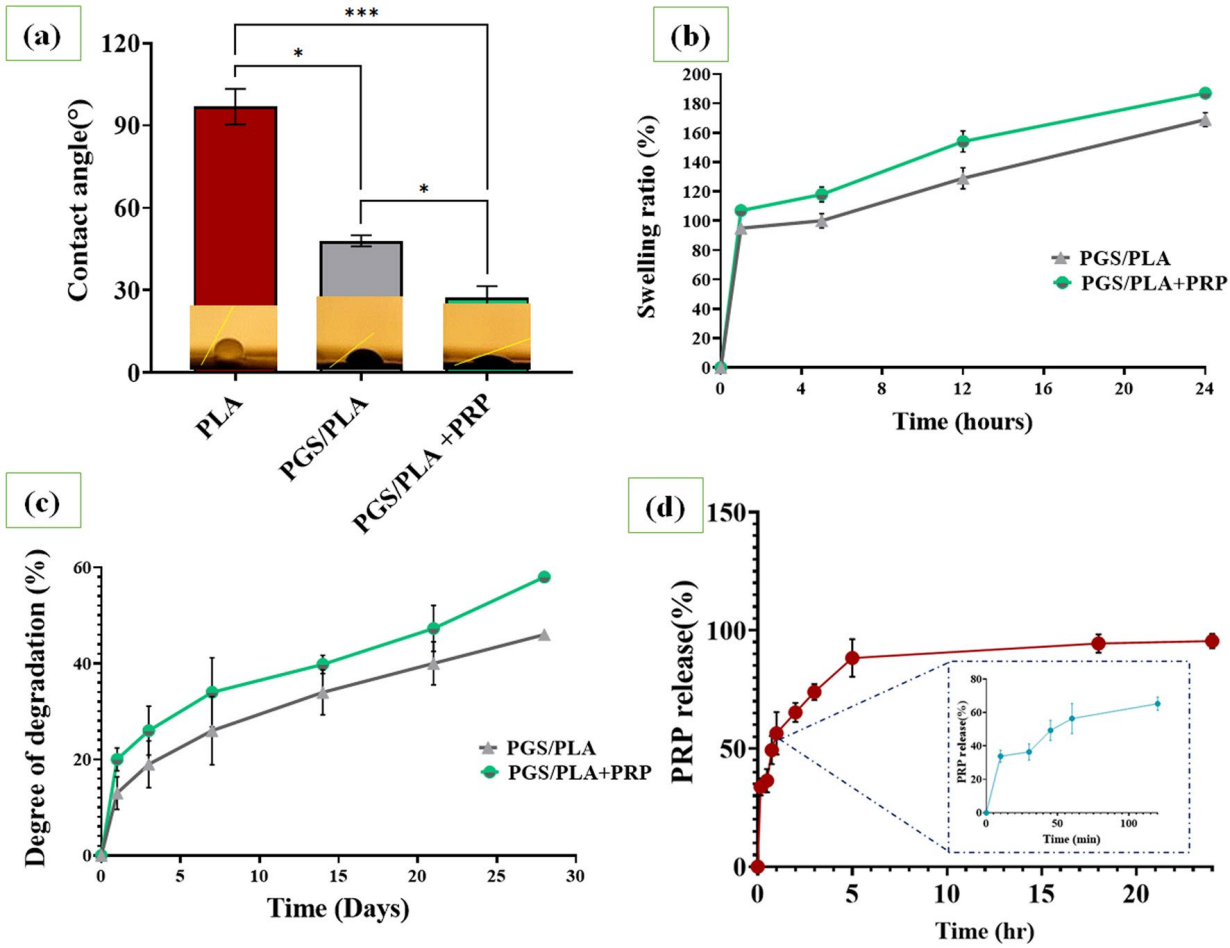
Physiological stability of PGS/PLA and PGS/PLA–PRP: (**a**) the water contact angle, (**b**) mass swelling ratio during 24 h of incubation in the PBS solution, (**c**) degradation of the scaffolds during 28 days of incubation in PBS, (**d**) evaluation of PRP release from PGS/PLA-PRP in the PBS solution at 37 °C during 24 h. All the values are expressed as means ± standard deviation for n = 3 specimens (*p < 0.05 and ***p < 0.001).

Using a water contact angle measurement, the wettability of PGS/PLA samples with or without PRP in various groups was investigated (Fig. 3a). The findings demonstrated that the addition of PGS in comparison to PLA scaffold considerably reduced the water contact angle of the PGS/PLA sample (p < 0.05). Significantly, the addition of PGS to the nanofiber structure increased the water contact angle from 96.2 ± 7° to 42.8 ± 2°. As previously reported in other research, the contact between the polymer chains and water molecules was improved by adding the PGS hydrophilic chemical groups, such as OH^30^. However, the water contact angle dramatically decreased (p < 0.05) with the addition of hydrophilic PRP composition. Significantly, with the addition of PRP, the water contact angle of PGS/PLA decreased from 42.8 ± 2° to 28.6 ± 5°. It is significant that the samples that had been processed with PRP displayed greater hydrophilicity, which may play an important role in cell responses such cellular adhesion and proliferation^41^.

An ideal wound dressing is one characterized by high swelling and absorption ability to absorb wound exudates and to keep wound moisture at the wound site for accelerated healing. As shown in Fig. 3b, the swelling ratios measured after 24 h for the PGS/PLA and PGS/PLA-PRP scaffolds were around 169 ± 4.7% and 187 ± 1.9%, respectively, with the swelling ratio of PGS/PLA-PRP scaffolds being slightly higher than that of PGS/PLA (p < 0.05). This is due to the presence of PRP with its hydrophilic functional groups (amine groups) that release the PRP present in the PBS solution to enhance penetration of water molecules into the structure. Clearly, both PGS/PLA and PGS/PLA-PRP exhibited ideal water swelling ratios (of more than 100%) for wound dressing applications^30^. Similarly, Melo et al.^42^ evaluated the swelling behavior of platelet-rich plasma (PRP)/hyaluronic acid (HA) and demonstrated by adding PRP to polymer structure the equilibrium ratio of swelling was increased due to presence PRP hydrophilic chemical groups and indicated that PRP/HA would be favorable for nutrient diffusion.

Degradation rate in the electrospun membranes was evaluated by immersing the specimens in the PBS solution for 28 days (Fig. 3c) to record in vitro degradation rates of 46 ± 1.2% and 57.9 ± 3% for PGS/PLA and PGS/PLA-PRP, respectively, after 28 days of treatment in PBS with pH ~ 7.4 (p ˂ 0.05). The difference observed between the measured degradation rates may be attributed to the higher hydrophilicity of the PGS/PLA-PRP composition. Moreover, PRP release from the membrane led to weight losses to create more empty spaces for water penetration whereby water radicals gained access to the polymer substrate, which led to an increase in the degradation rate. Similarly, Cheng et al.^43^ evaluated the degradation processes of silk fibroin (SF)/poly (ε-caprolactone) (PCL)—platelet-rich plasma (PRP) coaxial nanofiber and reported that by adding PRP to fibers, the degradation rate was increased due to hydrophilic structure and fast released of PRP. Moreover, maintaining the skin scaffold structure is an essential parameter in preserving the strength and the stress needed for skin tissue remodeling; hence, a controllable degradation profile is essential in wound dressings^44^.

### PRP release behavior

It is well established that the first 24 h after an injury is the critical period for the wound healing process as the growth factors released during this period seemingly control inflammation and accelerate wound healing and tissue regeneration^45,46^. In the present study, the cumulative amounts of PRP released from the PGS/PLA-PRP sample in PBS solution (Fig. 3d) showed a burst release (60%) during the first two hours while a sustained PRP delivery occurred over the next 20 h. The initial burst release may be due to surface-distributed PRP and degradation of PGS in PGS/PLA-PRP wound dressings^47,48^. Gomez et al.^46^ reported a similar trend for their 3D printed Carboxymethyl cellulose loaded with PRP. They showed that growth factors such as VEGF play an essential role in stimulating angiogenesis, cell migration, and collagen deposition during the first times of an ideal wound healing process^46^. According to a recent study by Diaz et al.^23^ on the biodegradable electrospun PCL scaffolds containing PRP, total protein and growth factors (PDGF, TGF-β, and VEGF) were released during a period of 4 days. However, compared to PCL or PLA nanofibers, the release in our study was faster. This is likely because PGS involved surface degradation behavior and PRP was more quickly released by PGS degradation in PBS environment^49^.

### In vitro cell assay

**Figure 4:**
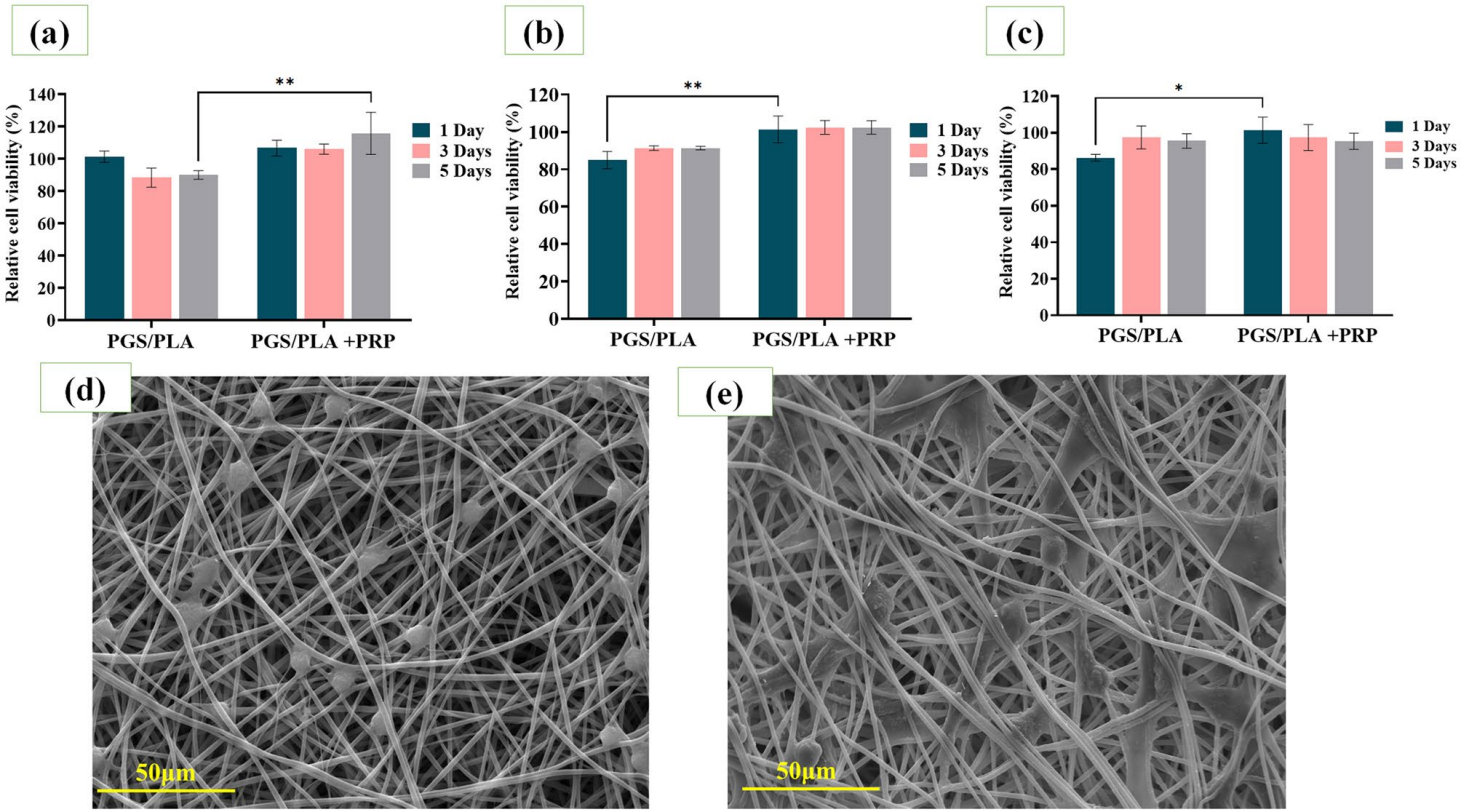
Cell cytotoxicity and cell adhesion evaluation: (**a**) L929 cell viability, (**b**) HUVEC cell viability, (**c**) RAW cell viability during 1, 3, and 5 days after culture, (**d**) SEM images of L929 cell morphology on the PGS/PLA-PRP, and (**e**) SEM images of HUVEC cell morphology on the PGS/PLA-PRP after 3 days. All the values are expressed as means (n = 3) ± standard deviation (*p < 0.05 and **p < 0.01).

Figure 4 presents cell viability and proliferation results obtained for L929, HUVEC, and RAW-264.7 cells against the PGS/PLA scaffolds in the absence and presence of PRP. The results indicate cytotoxicity during days 1, 3, and 5 for none of the three cell lines. Clearly, the viability of the L929 cells significantly increased during 5 days as a result of PRP addition to PGS/PLA (Fig. 4a). This may be attributed to the effects of the PRP growth factor release and the improved L929 proliferation cell culture assay. Similarly, Lucarelli et al.^50^ showed increased proliferation of stromal stem cells over a period of 6 days as a result of using 10%w/w PRP and burst release during first time.

According to Fig. 4b,c, the viability values measured for HUVEC and RAW cells demonstrated that PRP release improved macrophage cell growth and proliferation at day 1 (p ˂ 0.05) but that no significant differences in cell viability were detected between the PRP-containing and PRP-free specimens at days 3 and 5 (p ˃ 0.05). Moreover, the viability of RAW cells indicates a direct relationship between PRP release time and RAW cells proliferation^46^. Finally, it may be concluded that PGS/PLA is an ideal scaffold due to its cell viability of more than 85%. In the case of the PGS/PLA-PRP scaffold, not only did the presence of PRP not lead to cell toxicity but it also increased both cell growth and proliferation.

SEM images were examined to determine the behavior, adhesion, and morphology of L929 and HUVEC cell lines on PGS/PLA-PRP scaffolds after 3 days (Fig. 4d,e). According to Fig. 4d, the L929 cells attached to the surface of PGS/PLA-PRP and smeared well with a rounded morphology. On the other hand, the HUVEC cell attachment pattern (Fig. 4e) exhibited a flat and elongated morphology on the PGS/PLA-PRP surface with good cell adhesion to the specimen as judged from the cell surface. These ideal cell attachment patterns are due to the beneficial surface properties (such as porosity, water wettability, and morphology) and biological effect of the PRP-containing scaffolds that create a microenvironment favorable to cell bonding and cell adhesion. These findings are confirmed by Orue et al.^51^ who reported that skin dressing containing PRP showed a better cell viability, cell adhesion, and cell migration due to the beneficial properties of PRP.

### Scratch test (in vitro wound healing)

**Figure 5:**
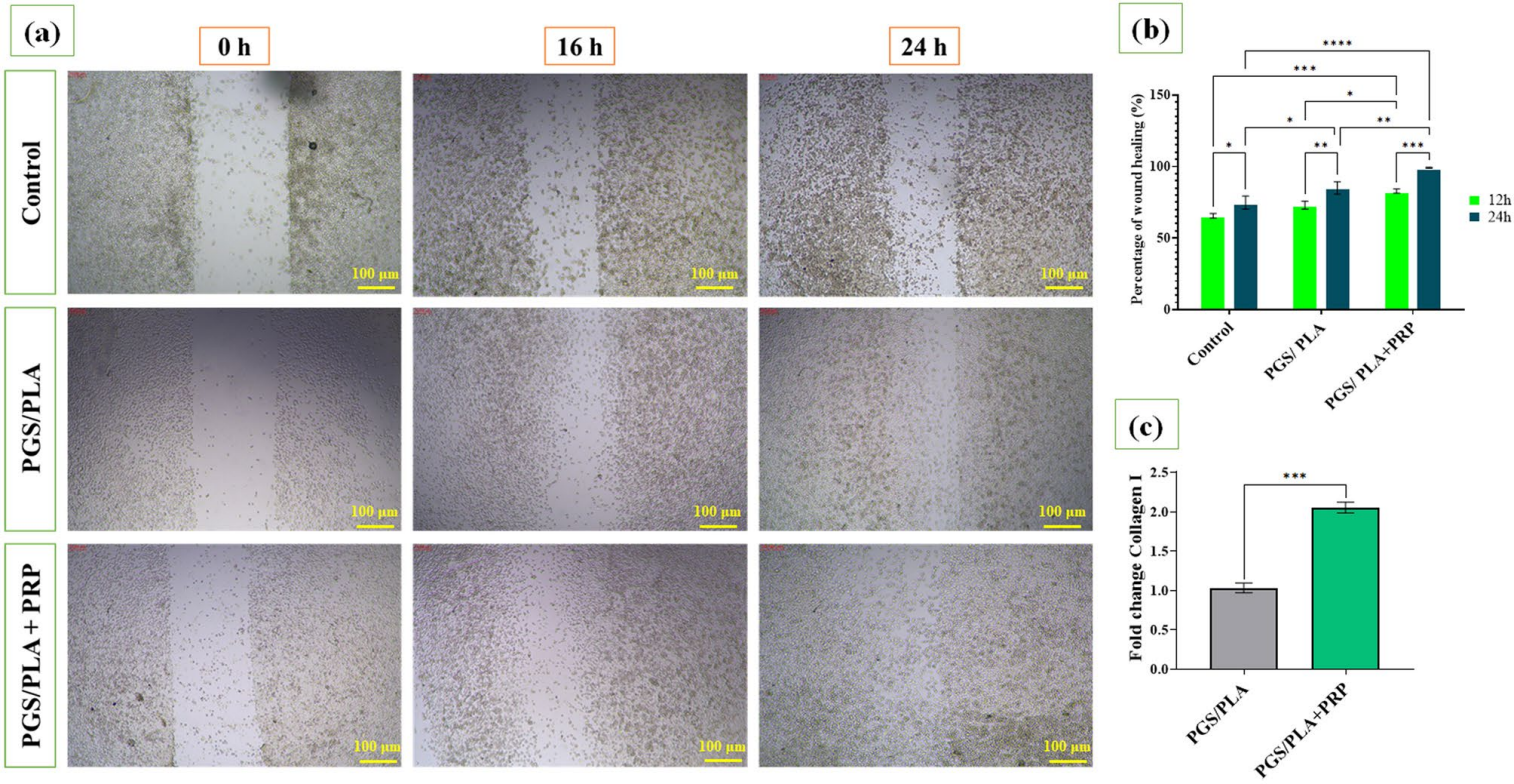
(**a**) In vitro wound healing of L929 cell proliferation, (**b**) wound healing rates expressed as percentages in the L929 scratch assay, and (**c**) relative expression of collagen I during 24 h. All the values are expressed as means (n = 3) ± standard deviation (*p < 0.05, **p < 0.01, ***p < 0.001, and ****p < 0.0001).

The scratch test was performed to investigate the effects of PGS/PLA on fibroblast cell migration and, thereby the wound healing process, in the absence/presence of PRP (Fig. 5). The results revealed no significant differences in scratch wound closure between the PGS/PLA and the control group after 12 h (p ˃ 0.05). However, PGS/PLA-PRP was observed to lead to significantly accelerated cell migration and wound healing after 12 and 24 h compared to the control (p ˂ 0.05). The assay showed wound healing percentages of 74.76 ± 6%, 85.06 ± 4%, and 99.05 ± 0.9% after 24 h for the control, PGS/PLA, and PGS/PLA-PRP specimens, respectively. The faster cell migration observed with PGS/PLA-PRP seemed to be due to the PRP release in 24 h as PDGF, VEGF, and other growth factors present in PRP were able to stimulate cell reaction to improve fibroblast cells migration^52^.

### Collagen I gene expression

It may be claimed that, during the wound healing process, fibroblast cells contribute mainly to collagen I production and deposition at wound sites^53^. In order to evaluate the effects of PGS/PLA and PGS/PLA-PRP on tissue remodeling during the wound healing process, the related collagen I gene expression was studied at the transcriptional level. As shown in Fig. 5c, the scaffold containing PRP promoted a higher collagen I expression (~ 2 times) over the 24-h period than did the PGS/PLA specimen, confirming the positive role of PRP in collagen production. Enhanced collagen synthesis and deposition in PGS/PLA-PRP could be due to the presence of PRP and its growth factors in the wound dressing^52^. Zhiyong et al.^54^ also reported that controlled and sustained release of PRP would accelerate skin collagen synthesis and deposition at the wound site. In another study, Zhang et al.^55^ designed an injectable, self-healing, and non-specific tissue-adhesive hydrogel based on chondroitin sulfate/carboxymethyl chitosan-PRP and demonstrated that by PRP delivery and its various growth factor the formation of granulation tissues, collagen deposition and angiogenesis were promoted. Similarly, Xu et al.^56^ examined the effect of autologous PRP on full-thickness wound healing and showed by adding PRP at wound site, the re-epithelialization, extra cellular matrix formation and synthesis collagen accelerated significantly.

### Inflammatory response

**Figure 6:**
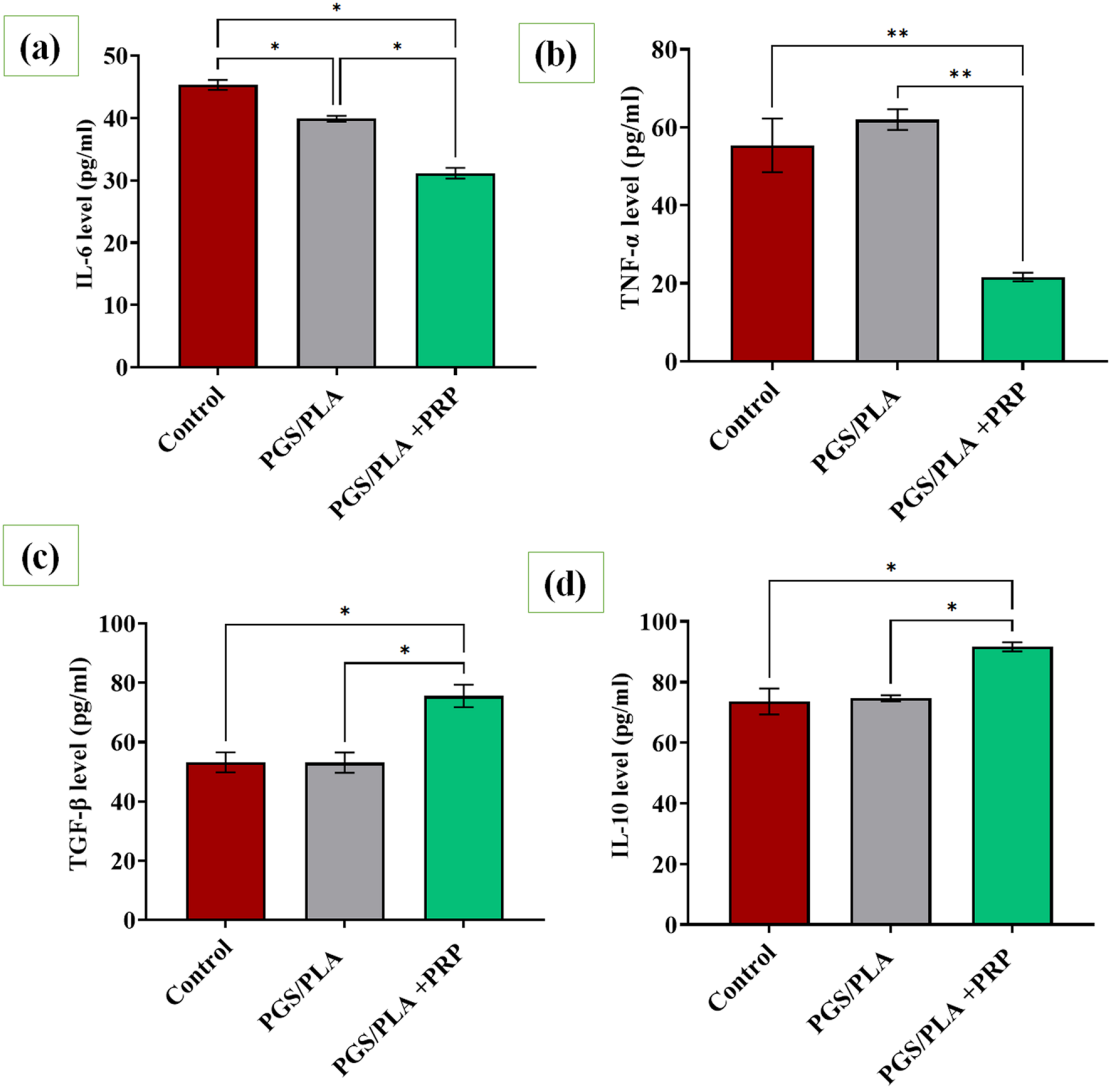
Anti-inflammatory effects of PGS/PLA and PGS/PLA-PRP as evaluated by RAW264.7 after 48 h of treatment: (**a**) IL-6, (**b**) TNF-α, (**c**) TGF-β, (**d**) IL-10. All the values are expressed as means (n = 3) ± standard deviation (*p < 0.05 and **p < 0.01).

Figure 6 presents the levels of pro-inflammatory (IL-6 and TNF-α) and anti-inflammatory (IL-10, TGF-β) parameters in LPS- stimulated cells seeded on PGS/PLA and PGS/PLA-PRP after 48 h of incubation. According to Fig. 6a,b, the highest values of IL-6 and TNF-α were observed in TCP and PGS/PLA in the absence of PRP, indicating that PGS/PCL was not able to reduce the expression levels of the pro-inflammatory cytokines. This is while the PGS/PLA structure, as compared with the PGS/PLA and control groups, exhibited reductions in pro-inflammatory mediators when PRP was added (p ˂ 0.05). This is evidenced by the TNF-α levels of about 55.3 ± 9, 61.98 ± 3, and 21.65 ± 2 µg/ml observed in the control, PGS/PLA, and PGS/PLA-PRP, respectively. Figure 6c,d demonstrate that the levels of cytokine (IL-10) and the growth factor (TGF-β) related to the anti-inflammatory phase (M2 state) were significantly higher in the PGS/PLA-PRP specimen than they were in the control and PGS/PLA groups (p ˂ 0.05). More specifically, TGF-β levels of around 53.15 ± 5, 51.2 ± 4, and 75.55 ± 6 pg/ml were recorded for TCP, PGS/PLA, and PGS/PLA-PRP specimens, respectively, indicating that PRP delivery promoted M2 macrophage polarization and led to increased expression levels of M2 macrophage mediators. Wei et al.^52^ also reported that adding PRP to the hydrogel enhanced the levels of anti-inflammatory factors (TGF-β and VEGF) but significantly inhibited those of pro-inflammatory factors (TNF-α, IL-1β, and IL-6).

### In vitro angiogenesis

**Figure 7:**
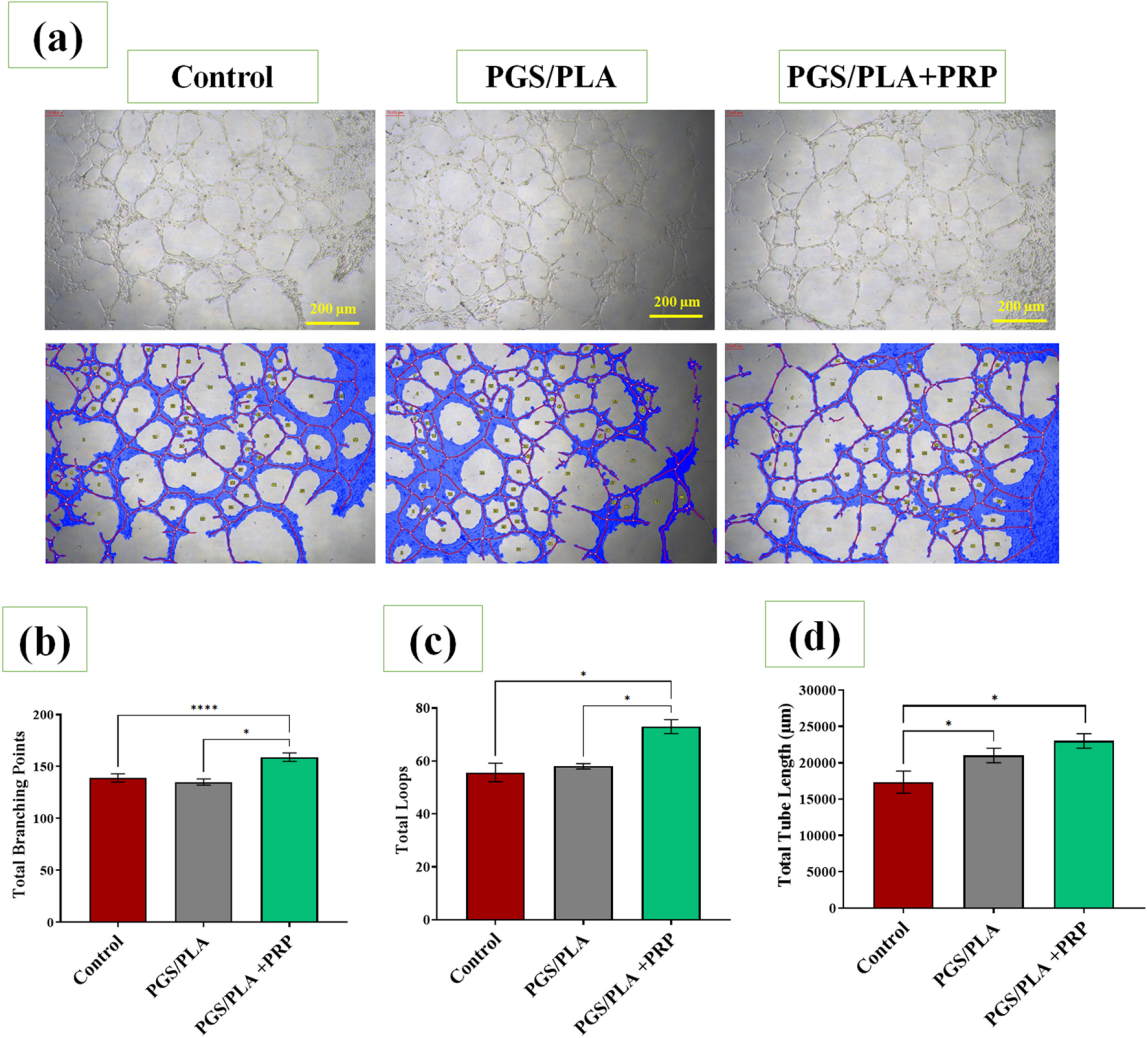
In vitro angiogenesis assay. (**a**) Digital images of tube formation by the HUVEC cell line in the Matrigel environment with PGS/PLA and PGS/PLA-PRP extracts for 24 h, (**b**) evaluation of total branching points, (**c**) total loops, and (**d**) total tube length. All the values are expressed as means (n = 3) ± standard deviation (*p < 0.05 and ****p < 0.0001).

The angiogenesis process was studied in TCP, PGS/PLA, and PGS/PLA-PRP scaffolds via HUVEC cells tube formation assay (Fig. 7). According to Fig. 7a, the HUVECs in all the three groups formed tube structures after 24 h. Moreover, the numbers of branching points and loops as well as tube lengths were higher in PGS/PLA-PRP than in the PGS/PLA and the control groups (Fig. 7b–e) as evidenced by the total branching points of 139 ± 4.9, 135 ± 3.1, and 159 ± 3.5 (Fig. 7a); total loops of 55.66 ± 6, 58.1 ± 2, and 73.4 ± 3 (Fig. 7b); and total tube lengths of about 17,333.3 ± 2967 μm, 21,500.6 ± 1900 μm, and 22,590.9 ± 1780 μm (Fig. 7c) obtained for the control, PGS/PLA, and PGS/PLA-PRP groups, respectively. In general, there is a significant difference in total branching points and total loops due to the addition of PRP compared to the control sample and PGS/PLA, and the number of vessels has increased dramatically. It might, therefore, be claimed that the proper release and sustained activity of PRP in the PGS/PLA-PRP scaffold increased angiogenesis. This can be attributed to the presence of such growth factors in PRP as VEGF^57^. This is confirmed by Zheng et al.^49^ who maintained that angiogenesis and cell migration accelerated as a consequence of the release of PRP and its related growth factors so that the wound would heal with less scarring.

Our results revealed that the PGS/PLA-PRP wound dressing could be suitable candidate for skin tissue engineering and wound healing application, according to physical and biological activity. Moreover, the incorporation of PRP to structure of PGS/PLA membrane resulted in improved cell viability, cell migration, Enhanced inflammation responses, promoted angiogenesis and collagen deposition for ECM formation.

## Conclusion

To fabricate a new generation of biomaterial wound dressings, PRP was extracted from human blood, PGS was synthesized, and the two types of PGS/PLA and PGS/PLA-PRP fiber wound dressings were fabricated by electrospinning. It was observed that PRP addition not only affected the viability, growth, migration, proliferation, and adhesion of L929, RAW, and HUVEC cells but it also improved water absorption, biodegradation, collagen synthesis, antigenicity potential, and controlled inflammation of the scaffolds made. Moreover, enhancements were observed in the porosity, swelling ratio, and biodegradability of the scaffolds containing PRP when compared with the plain PGS/PLA ones. Another finding of the study revealed that the PGS/PLA-PRP specimens released PRP during 24 h of in vitro incubation. Also, cell assessments indicated the greater viability, proliferation, migration, and adhesion of the PGS/PLA-PRP scaffolds relative to those measured in the plain PGS/PLA scaffolds. The presence of PRP in the PGS/PLA-PRP scaffolds led not only to enhanced collagen deposition and angiogenesis but also to controlling inflammation factors involved in wound healing. It was concluded that the PGS/PLA-PRP scaffold is capable of stimulating skin regeneration, which makes it a desirable candidate for use in wound dressing application. However, a more detailed examination of the effect of PRP release from the PGS/PLA electrospun scaffold on wound repair in an *In-vivo* study should be performed.

## Data Availability

The datasets generated and analyzed during the current study are available from the corresponding author on reasonable request.
